# Solving characteristic parameters of heavy-duty gas turbines using parameter estimation method

**DOI:** 10.1371/journal.pone.0333661

**Published:** 2025-10-08

**Authors:** Jinling Chi, Chang Wang, Yangxue He, Chenxu Gou, Zhao Wang

**Affiliations:** 1 School of Mechanical and Electrical Engineering, China University of Mining and Technology (Beijing), Beijing , China; 2 Key Laboratory of Intelligent Mining and Robotics, Ministry of Emergency Management, Beijing, China; Indian Institute of Technology Kanpur, INDIA

## Abstract

In gas turbine simulation, precise parameterization of components is essential for reliable performance prediction, yet manufacturers usually provide only limited operational data. To address this issue, this study proposes a modeling approach based on limited operational parameters and applies it to a 9FA heavy-duty gas turbine. The framework employs maximum likelihood parameter estimation within an inverse problem formulation, combined with a modular methodology to reconstruct compressor characteristic curves and establish a full-condition mathematical model. The maximum relative error between the predicted and actual values for the discharge flow rate and discharge temperature of the compressor under steady-state standard conditions is no greater than 0.2%. Simulation results show that the relative errors for compressor isentropic efficiency and combustion efficiency are 0.34% and 0.1%, with parameter prediction errors below 0.5%. The relative errors for combustion pressure loss coefficient is 2.86%. Additional cross-validation using inverse problem methods further confirms the accuracy of the proposed approach under limited data conditions. These findings demonstrate that the method provides a valuable approach for full-condition gas turbine modeling and performance analysis.

## 1 Introduction

As an important energy power device, gas turbines play a crucial role in carbon emission reduction and renewable energy integration [1,2]. They are also core components of future energy systems, such as IGCC and near-zero emission power plants. Therefore, establishing gas turbine models, conducting performance analysis, and predicting full operating condition performance are of great significance for developing operational models and control strategies for gas turbines [3–5].

The key to obtaining accurate gas turbine performance lies in establishing mathematical models that align with component performance characteristics and setting the correct key parameters. A gas turbine consists of three main components: the compressor, combustor, and turbine, with the compressor being the core component. The accuracy of compressor characteristic map calculation directly determines the reliability of the overall simulation. Consequently, compressor performance prediction has always been a major research focus.

In practical gas turbine performance simulation [6], existing advanced modeling methods (such as neural network approaches [7–9] and polynomial fitting techniques [10,11]) generally rely on large training datasets, with most studies focusing on algorithmic optimization under the assumption that complete component characteristic maps are available. However, in real applications, users often lack access to complete characteristic data for core components like compressors, and can usually obtain only limited operating data or steady-state condition parameters (e.g., efficiency, pressure ratio, and temperature). How to reconstruct complete and accurate compressor characteristic maps from such limited data remains an unsolved problem. Essentially, this task belongs to the class of inverse problems, where sparse data points must be extrapolated into a full characteristic curve, which remains a weak point in current research.

When only limited experimental data are available, there is still no systematic method for inferring component performance parameters. Many scholars have proposed approaches for compressor characteristic map prediction, including polynomial fitting, elliptical equation fitting [12–17], modular extrapolation [18,19], and statistical methods [20–22]. However, because of the strong coupling among critical parameters (such as efficiency and characteristic equation coefficients [23–25]), traditional characteristic-equation-based methods often lead to single-parameter optimization, cumulative error, and distortion in results. Existing research also shows evident limitations of both classical and intelligent algorithm-based methods. For example, Fahrmeir [26] proposed a linear model for compressor characteristic curve estimation, but it exhibits poor adaptability to sparse data; Zhang Ye [27] applied traditional polynomial fitting with least squares, yet the accuracy of the resulting maps was relatively low; Song Zhaoyun [28] employed genetic algorithms for loss minimization, but the approach was prone to overfitting under small-sample conditions; Huang Wei [29] used MEA-BP neural networks to predict compressor characteristics, though the method suffered from weak generalization and high error under unseen operating conditions; Ying Yulong [30]’s iterative algorithm required more than 500 iterations per run, making it inefficient for real-time simulation; Afila [31] applied a PINN-based method, but error propagation became significant in complex multi-component coupled systems.

In contrast, studies on maximum likelihood estimation (MLE) have demonstrated certain advantages. Fisher [32] and Dempster [33] emphasized that the joint maximization of the likelihood function allows global optimization across multiple parameters, which fundamentally differs from traditional parameter-by-parameter adjustment approaches. Bai [34] further applied MLE to model estimation, showing that MLE possesses favorable small-sample properties, thus providing a theoretical basis for its application. Nevertheless, MLE has not yet been integrated with modular modeling of gas turbines, and its application to inverse prediction of heavy-duty gas turbine characteristics remains limited. Therefore, developing a global optimization framework capable of jointly estimating multiple critical parameters is essential for improving model accuracy, and this represents a systematic challenge that remains unsolved.

To address this issue, this paper proposes an inverse-solving method for heavy-duty gas turbines based on maximum likelihood parameter estimation. By combining modular modeling [35] with MLE, the method avoids error accumulation and single-parameter optimization, achieving multi-parameter global optimization. It enables the reconstruction of complete compressor characteristic maps from sparse data points, overcoming the limitations of linear models and data-dependent intelligent algorithms. A full gas turbine operating model is then developed, and comprehensive performance simulations are conducted. Through inverse solving, steady-state standard condition data are obtained, critical performance parameters and model parameters are predicted, and the results are validated against actual operating data. This establishes a closed-loop framework from parameter estimation to operating condition verification.

The study focuses on the GE 9351FA gas turbine deployed at a power plant in Beijing, which is widely used in China as an F-class gas turbine. Long-term operating data accumulated at the plant enhance both the practical relevance and engineering value of this research. Subsequent verification shows that the predicted results closely match the experimental data, confirming the effectiveness and consistency of the proposed method.

Sect 2, the literature and theoretical background of parameter estimation methods are reviewed in detail. Sect 3 presents the modeling of the gas turbine components and identifies the important parameters to be estimated in the model. Sect 4 employs a modular and maximum likelihood estimation method for predicting compressor characteristic curves, and the data calculated by the new characteristic curve is compared with the experimental data for error analysis. Sect 5, the full working condition model of the gas turbine is established, the full working condition simulation is carried out, the parameters obtained by the inverse solution are used to predict the gas turbine performance, and the results are compared with the actual operation data to verify the accuracy and effectiveness of the method. Sect 6 summarizes the conclusions.

## 2 Maximum likelihood parameter pstimation methods

Parameter estimation is the process of adjusting the parameters of a model so that the computed results of the system align with actual values. This method has gained widespread application across various fields due to its advantages, such as the ability to simultaneously analyze steady-state and dynamic model data, handle multiple parameters within these models, and the extensive statistical validation it has.

The parameter estimation method employed in this study is based on the maximum likelihood equations(MLE). This approach allows for the estimation of both the parameters of the physical model itself and the variance model parameters, which include the constant variance, constant relative variance, and heterogeneous variance of the experimental measurement data. During the process of solving the maximum likelihood equations(MLE), the parameters of the physical and variance models are determined based on the principle that the mathematical model should most accurately conform to the experimental measurements. The specific steps of the maximum likelihood-based parameter estimation method are as follows.

### 2.1 Objective function

It is assumed that the measurement error, mean and standard error of independent distribution and normal distribution are 0, the maximum likelihood objective can be obtained by the following objective Eq 1:

$$\Phi =\frac{N}{2}ln2\pi +\frac{1}{2}min_{\theta }\left\{\sum_{i=1}^{N_{E}}\sum_{j=1}^{N_{v_{i}}}\sum_{k=1}^{N_{M_{ij}}}\left[ln\left(\sigma_{ijk}^{2}\right)+\frac{{\left(\tilde{Z_{ijk}}-Z_{ijk}\right)}^{2}}{\sigma_{ijk}^{2}}\right]\right\}$$ (1)

Where:

N: The total number of measurements across all experiments;

*θ*: Parameter under estimation ($\theta_{L}$<*θ*<$\theta_{U}$);

i: Number of experiments (i=[1, *N*_*E*_]);

$N_{V_{i}}$: The number of variables measured in the i-th experiment;

$N_{M_{ij}}$: The number of measurements of variable j in the i-th experiment;

$\sigma_{ijk}^{2}$: The variance of the k-th measurement of variable j in the i-th experiment;

$\tilde{Z_{ijk}}$: The k-th measurement value of variable j in the i-th experiment;

*Z*_*ijk*_: The k-th model prediction of variable j in the i-th experiment.

### 2.2 Optimization algorithm

The Non-Linear Programming (NLP) problem is solved using a Sequential Quadratic Programming (SQP) algorithm with a maximum likelihood approximation (MLE). Assume the minimum length of linear search step is set to 10^−5^. The convergence criterion is defined by the following Eq 2:

$$\frac{1}{\left|\Phi^{*}+1\right|}\left(\left|\sum_{j}\frac{\partial \Phi^{*}}{\partial \theta_{j}}\delta \theta_{j}\right|+\sum_{j}\left|\mu_{j}\right|max\left(0,\theta_{j}^{L}-\theta_{j}^{*},\theta_{j}^{*}-\theta_{j}^{U}\right)\right)\;\;+\sum_{j}max\left(0,\theta_{j}^{L}-\theta_{j}^{*},\theta_{j}^{*}-\theta_{j}^{U}\right)\leq \varepsilon$$ (2)

Where:

*ε*: Optimization tolerance;

$\theta_{j}^{*}$: The j-th parameter to be estimated (including model parameters and variance model parameters);

$\theta_{j}^{*}$: The final value of parameter $\theta_{j}$;

$\theta_{j}^{U}$: The upper bounds of $\theta_{j}$ respectively;

$\theta_{j}^{L}$: The lower bounds of $\theta_{j}$ respectively;

$\Phi^{*}$: The final value of the maximum likelihood objective function;

$\partial \theta_{j}$: The step size of $\theta_{j}$ in the last iteration of the parameter estimation;

$\mu_{j}$: The Lagrange multiplier associated with the boundary constraint of $\theta_{j}$.

### 2.3 Results validation

Validation of the parameter estimation results involves two aspects: statistical t-test and goodness-of-fit test.

(1) Statistical t-test:

The t-value of the estimated parameters from the process model and variance model, is calculated by $t_{i}=\hat{\theta_{j}}/X_{i}\left(0.95\right)$, which represents the reliability of the estimated values within a 95% confidence interval. Comparing these t-values with a reference t-value t(0.95, N-$N_{\theta }$), when a t-value is greater than the corresponding reference value, it prove that the estimation of the respective parameter is accurate. When it is less than the reference value, it suggests lower precision in the estimation of the respective parameter.

(2) Goodness-of-fit test:

Based on the goodness-of-fit test, the comparison of the weighted sum of squared residuals, as shown in Eq 3:

$$\sum_{i=1}^{N_{E}}\sum_{j=1}^{N_{v_{i}}}\sum_{k=1}^{N_{M_{ij}}}\left[\frac{{\left(\tilde{Z_{ijk}}-Z_{ijk}\right)}^{2}}{\sigma_{ijk}^{2}}\right]$$ (3)

With a certain confidence level *x*^2^ determines whether the results meet the required criteria. If the weighted residuals exceed the *x*^2^ value, the fit fails, indicating that the model cannot accurately reflect the physical system.

## 3 Gas turbine unit models and parameters to be estimated

This paper establishes mathematical models for key components of a heavy-duty turbine about the compressor, combustion chamber and turbine based on gPROMS [36], and identifies important parameters to be estimated in each model.

### 3.1 Compressor model

When simulating a gas turbine, there are two approaches for modeling the compressor: the compressor model method based on characteristic curves and the compressor model method based on characteristic equations.

#### 3.1.1 Compressor characteristic curve model.

The characteristic curve of the compressor depicts the relationship between pressure ratio and efficiency during actual operation under different ambient conditions such as atmospheric temperature and pressure, varying with speed and flow rate [37,38]. In the compressor characteristic curve model, based on similarity theory, non-dimensional similar parameters are utilized to ensure that the universal compressor characteristic curve is applicable under different intake conditions. By non-dimensionalizing the universal compressor characteristic curve information and representing it in matrix form, interpolation is used to compute the operational points along non-dimensional similar speed lines and compressor characteristic curve variable lines. This method accurately reflects the actual operation of the compressor corresponding to the characteristic curve.

#### 3.1.2 Compressor characteristic equation model.

In the vast majority of cases, the characteristic curve of a compressor is unknown. The characteristic equation model is established based on the analysis of the characteristic curve and then appropriately simplified. In the characteristic equation model, linear equations can be used to replace the compressor characteristic curve. Temperature, speed, and pressure correction coefficients are used to modify the simplified equations appropriately, as shown in Eqs (4,5,6). When these correction coefficients are set appropriately, the characteristic equation model can also effectively reflect the operational characteristics of the compressor.

The relationship between compressor inlet flow rate, temperature, and pressure is as follows:

$$q_{m}P_{d}Tr_{d}=q_{m,d}PT_{d}r\left(1-a_{TFC}\frac{T-T_{d}}{T_{d}}\right)\left(1-b_{PFC}\frac{\pi -\pi_{d}}{\pi_{d}}\right)$$ (4)

Where:

*q*_*m*_: Actual air mass flow rate at the compressor inlet, kg/s;

*P*: Actual air pressure at the compressor inlet, Pa;

*T*: Actual air temperature at the compressor inlet, K;

*r*: Actual compressor speed, r/min;

*π*: Actual compressor pressure ratio;

*q*_*m*,*d*_: Compressor inlet air mass flow at design point;

*P*_*d*_: Compressor inlet air pressure at design point;

*T*_*d*_: Compressor inlet air temperature at design point;

*r*_*d*_: Compressor inlet air speed at design point;

$\pi_{d}$: Compressor inlet air pressure ratio at design point;

*a*_*TFC*_: The temperature correction coefficient for the flow rate;

*b*_*PFC*_: The pressure ratio correction coefficient for the flow rate.

The relationship between the compressor isentropic efficiency and non-dimensional similar speed is as follows:

$$\eta_{c}=\eta_{max}\left(1-c_{NEC}\frac{\left|\overset{―}{r}-{\overset{―}{r}}_{\eta ,max}\right|}{\overset{―}{r_{\eta ,max}}}\right)\left(1-d_{PEC}\frac{\left|\pi -\pi_{d}\right|}{\pi_{d}}\right)$$ (5)

$$\eta_{c,d}=\eta_{max}\left(1-c_{NEC}\frac{\left|\overset{―}{r}-{\overset{―}{r}}_{\eta ,max}\right|}{{\overset{―}{r}}_{\eta ,max}}\right)$$ (6)

Where:

$\eta_{c}$: Actual isentropic efficiency of the compressor;

$\eta_{max}$: Maximum isentropic efficiency of the compressor;

$\overset{―}{r}$: The dimensionless similar speed;

${\overset{―}{r}}_{\eta ,max}$: The dimensionless similar speed corresponding to the maximum efficiency;

*c*_*NEC*_: The speed correction factor for efficiency;

*d*_*PEC*_: The pressure ratio correction factor for efficiency.

The isentropic efficiency at the compressor design point and the correction factors *a*_*TFC*_, *b*_*PFC*_, *c*_*NEC*_, *d*_*PEC*_, which reflects the characteristics of different compressor characteristic curves,can be obtained through parameter estimation using experimental data.

### 3.2 Combustion chamber model

The combustion chamber model is established based on the principle of energy conservation. Based on combustor efficiency, considering unburned fuel losses and heat losses in the combustor. The pressure loss in the combustion chamber reflects the inlet and outlet pressure losses, as shown in Eqs (7,8) [39]:

$$\eta_{comp}=\frac{m_{fuel}*LHV_{fuel}-h_{loss}}{m_{fuel}*LHV_{fuel}}$$ (7)

$$P_{drop}=\frac{P_{out}-P_{in}}{P_{in}}$$ (8)

In the above two equations:

$\eta_{comp}$: On efficiency;

*m*: Flow rate;

*h*: Enthalpy;

*P*: Pressure;

*fuel* : Fuel;

*loss*: Loss value;

*in*: Combustor inlet conditions;

*out*: Combustor outlet conditions.

The combustion chamber efficiency and pressure loss can be estimated using experimental data.

### 3.3 Turbine model

The off-design operation of the turbine is calculated using the relationship of the first-stage turbine nozzle under choked conditions as given by Eq 9, which defines the relationship between turbine inlet flow rate, temperature, pressure, and nozzle area, assuming that the turbine nozzle area is a constant fixed value. During variable conditions calculation, the nozzle area and turbine inlet conditions (flow rate, temperature) derived from the design conditions are used to calculate the turbine first-stage nozzle inlet pressure under choked conditions.

$$\frac{W\sqrt{T_{in}}}{A_{nozzle}p_{in}}=\sqrt{\frac{\gamma }{R}{\left(\frac{2}{\gamma +1}\right)}^{\frac{\gamma +1}{\gamma -1}}}$$ (9)

Where:

*W*: Nozzle inlet flow rate;

*A*_*nozzle*_: Nozzle area, which is constant under both design and off-design conditions;

*T*_*in*_: Inlet temperature;

*p*_*in*_: Inlet temperature and pressure, respectively;

*R*: Gas constant;

*γ*: Specific heat ratio.

In the turbine cooling model, the amount of cooling air under varying turbine conditions is considered. Based on the design values, adjustments are made according to the temperature and pressure at the extraction point:

$$\dot{m_{c}}={\dot{m}}_{c_{des}}\left(\frac{P_{c}}{P_{c_{des}}}\right){\left(\frac{T_{c_{des}}}{T_{c}}\right)}^{0.5}$$ (10)

Where:

$\dot{m_{c}}$: Flow rate of the cooling model;

${\dot{m}}_{c_{des}}$: Flow rate at the design point of the cooling model;

*P*_*c*_: Pressure under the cooling model;

$P_{c_{des}}$: Pressure at the design point of the cooling model;

*T*_*c*_: Temperature under the cooling model;

$T_{c_{des}}$: Temperature at the design point of the cooling model.

In the process of mixing cooling air with hot gas, the acceleration of cooling air to match the velocity of hot gas results in a decrease in the velocity of the hot gas. This loss can be quantified as the pressure loss of the hot gas calculated by Eq 10, which can be incorporated into the pressure loss of the combustion chamber or translated into an impact on the variable efficiency of the turbine. In this study, the model considers the variable efficiency loss of the turbine. Eqs (11,12,13) calculate the turbine’s variable efficiency loss caused by this pressure loss. The blending loss parameter ζ represents the loss due to the different directions of injected air and hot gas. In the model, ζis combined with the specific heat ratio *k*_*g*_ and the Mach number *M*_*g*_ into a single parameter *K*. For most air turbines, the specific heat ratio *k*_*g*_ is approximately 1.3, the Mach number *M*_*g*_ ranges from 0.6 to 0.8, and ζ is roughly between 0.3 and 0.6. Therefore, the range of parameter *K* should be between 0.15 and 0.5.

$$\frac{\Delta p}{p_{tbn,inlet}}=-\frac{{\dot{m}}_{c}}{{\dot{m}}_{g}}\kappa_{g}M_{g}^{2}\zeta =-\frac{{\dot{m}}_{c}}{{\dot{m}}_{g}}K;\Delta p<0$$ (11)

$$\frac{\eta_{p,uc_{tbn}}+\Delta \eta }{\eta_{p,uc_{tbn}}}=\frac{ln\left(p_{tbn,exit}/p_{tbn,inlet}\right)}{ln\left(p_{tbn,exit}/\left(p_{tbn,inlet}+\Delta p\right)\right)}$$ (12)

$$\eta_{p,c_{t}bn}=\eta_{p,uc_{tbn}}-\Delta \eta ;\Delta \eta >0$$ (13)

The amount of cooling air and the parameter *K* under design conditions can be estimated through experimental data.

## 4 Extrapolation of compressor characteristics curves based on modeling method

In this study, the PG9351FA gas turbine compressor characteristic curve was estimated using performance data provided by GE under design conditions, along with experimental data from non-design operating points of the same model unit at a thermal power plant in Beijing. The optimal modeling factors are obtained using the maximum likelihood parameter estimation method to derive a new compressor characteristic curve.

The experimental data used corresponds to the IOS design condition performance of the 9FA compressor, as shown in Table 1.

**Table 1:** The design condition performance of the PG9351FA gas turbine.

Pressure ratio	Inlet air mass flow	Exhaust temperature
16.576	647.96kg/s	680.48K

### 4.1 Modeling method and parameters to be estimated

**Fig 1:**
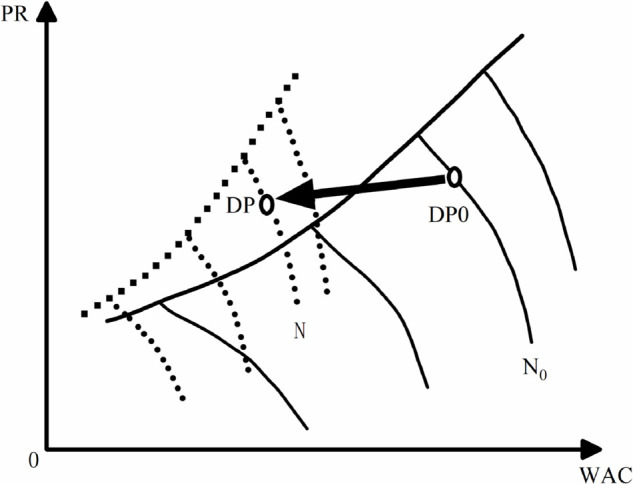
Correction of the compressor characteristic curve under design conditions.

The modeling process under design condition is depicted in Fig 1 :

The most critical step in modeling is to find the optimal modeling factors, which include design condition modeling factors and off-design condition modeling factors. In the design condition modeling factors, *NPR* = *NCF* = *NEF* = 1 is chosen as the central reference point for the modeling process. The position of any point on the new characteristic curve relative to the original characteristic curve is determined by the modeling factors and the distance of that point from the central reference point.

The original characteristic curve is dimensionless. Therefore, the characteristic curves of the design points are adjusted using the new design point data of the 9FA. The new characteristic curves for speed, pressure ratio, flow rate, and efficiency are given by Eqs (14,15,16,17):

$$N=N_{0}$$ (14)

$$PR=NPR\cdot \left(PR_{des}-1\right)+1$$ (15)

$$CF=NCF\cdot CF_{des}$$ (16)

$$EF=NEF\cdot EF_{des}$$ (17)

NPR, NCF, NEF represent dimensionless points on the original characteristic curve, with subscript “des" indicating the design point data for the new 9FA characteristic curve. PR, EF, and CF denote the pressure ratio, efficiency, and flow rate at corresponding points on the new characteristic curve.

**Fig 2:**
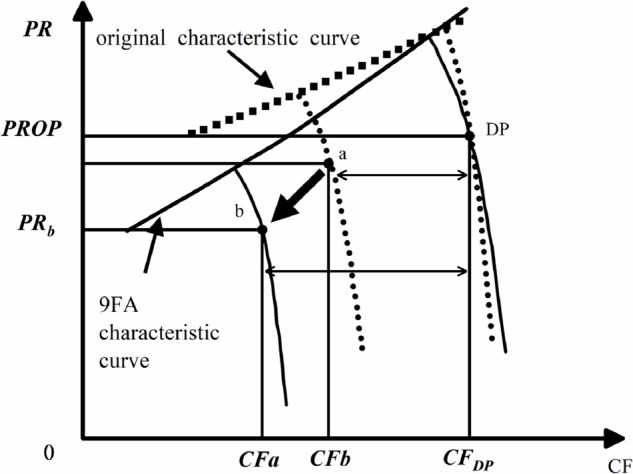
Compressor characteristic curve change correction.

The modeling process under off-design conditions is depicted in Fig 2:

The off-design modeling factors directly relate off-design conditions to design conditions. Due to changes in environmental conditions, the characteristics of the compressor parameters can change. Therefore, a dimensionless compressor characteristic curve that considers the influence of environmental conditions is used, where CF represents the relative equivalent flow rate. This is the ratio of the equivalent flow rate at any point on the characteristic curve to the equivalent flow rate at the design point. Similarly, there are relative equivalent pressure ratio NPR and relative equivalent efficiency NEF. The modeling factors for equivalent flow rate, pressure ratio, and isentropic efficiency are defined by Eqs (18,19,20):

$$SF_{CF,OD}=\frac{\left(CF_{b}-CF_{DP}\right)}{CF_{a}-CF_{DP}}$$ (18)

$$SF_{EF,OD}=\frac{\left(EF_{b}-EF_{DP}\right)}{EF_{a}-EF_{DP}}$$ (19)

$$SF_{PR,OD}=\frac{\left(PR_{b}-PR_{DP}\right)}{PR_{a}-PR_{DP}}$$ (20)

Where DP represents the design operating point selected as the reference point on the default original characteristic curve, it remains fixed during adjustments to non-design conditions. Point a denotes any point on the original characteristic curve before adjustments deviate from design conditions, while point b represents the corresponding point of point a on the new characteristic curve after modularization under non-design conditions. During adjustments for off-design conditions, corrections were also applied to the rotational speed, but since the correction factor chosen is equal to 1, the corrected speed remains identical to the speed on the original characteristic curve. With these defined correction factors, any point on the original characteristic curve can be matched to its corresponding point on the new characteristic curve.

### 4.2 Results of characteristic curve modeling

The experimental data consists of several sets of corresponding exhaust pressure, exhaust temperature, and exhaust flow data at a relative speed of 1.00 with constant IGV angle, and at relative speeds of 81, 87, 94.6, 97.1, 100, 102, and 106.2 with varying IGV [40]. Using the maximum likelihood estimation method and the approach for determining optimal modeling factors described in Sect 3, the design condition modeling factors and off-design condition modeling factors were computed. The results for each dataset are presented in Table 2.

**Table 2:** Off-design modeling factors for characteristic curves at various corrected speeds.

	CS=81	CS=87	CS=94.6	CS=97.1	CS=100	CS=102	CS=106.2
*SF*_*PR*,*OD*_	0.42864	0.50427	0.69927	0.84457	1.08605	1.24009	1.31868
*SF*_*CF*,*OD*_	2.65089	3.15588	1.93341	1.48862	1	1.343128	0.14174

**Fig 3:**
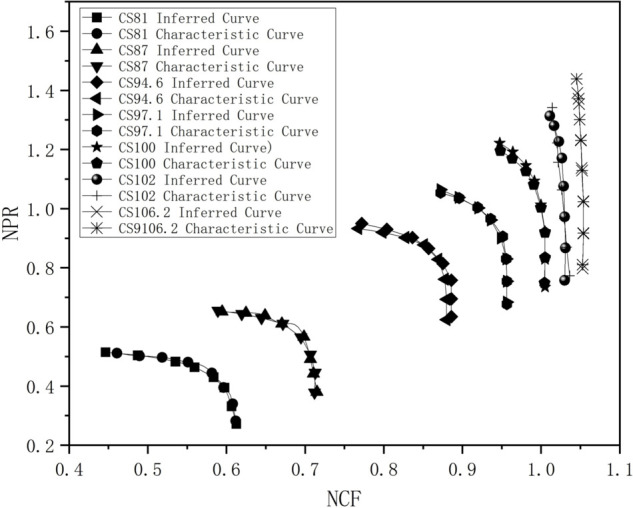
Results of modulization method.

Fig 3 compares the inferred new characteristic curve obtained through modularization with the given original characteristic curve. From the figure, it is evident that this parameter estimation-based modularization method produces a characteristic curve that better matches the original characteristic curve.

**Fig 4:**
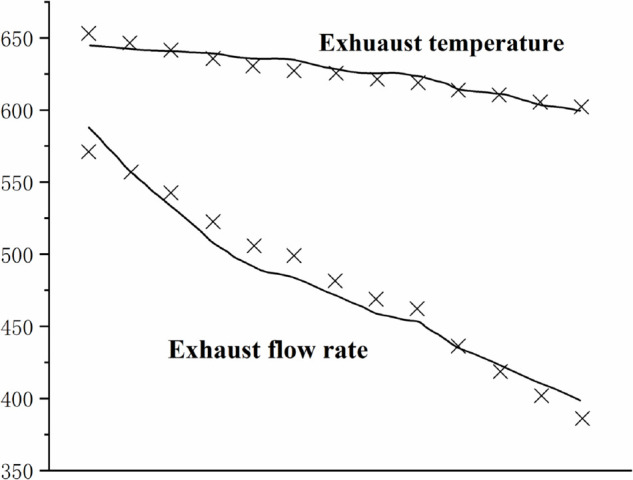
Estimated results when CS=0.946.

**Fig 5:**
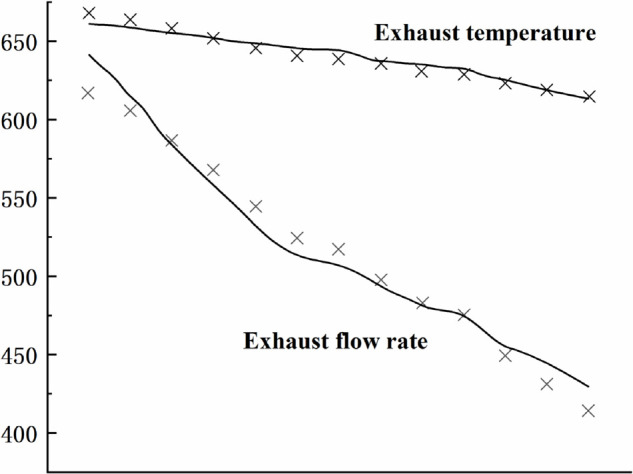
Estimated results when CS=0.971.

**Fig 6:**
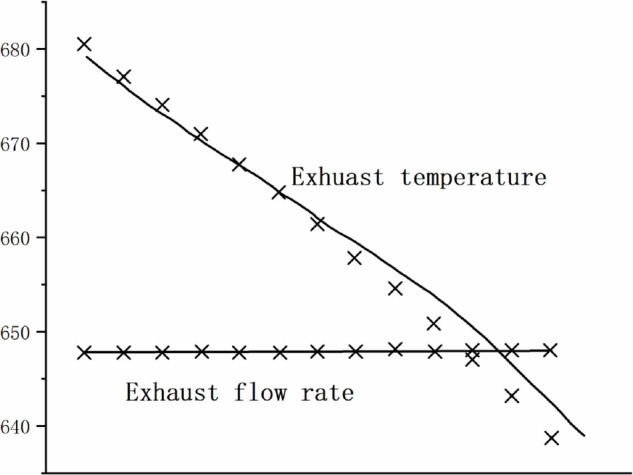
Estimated results when CS=1.00.

**Fig 7:**
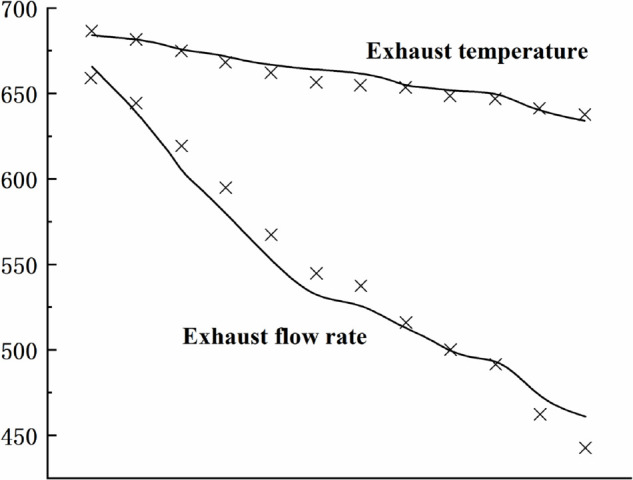
Estimated results when CS=1.02.

Fig 4,5,6,7,8 shows the comparison between the exhaust flow rate and exhaust temperature values obtained from calculations using the new characteristic curve at various corrected speeds, and the experimental data.

**Fig 8:**
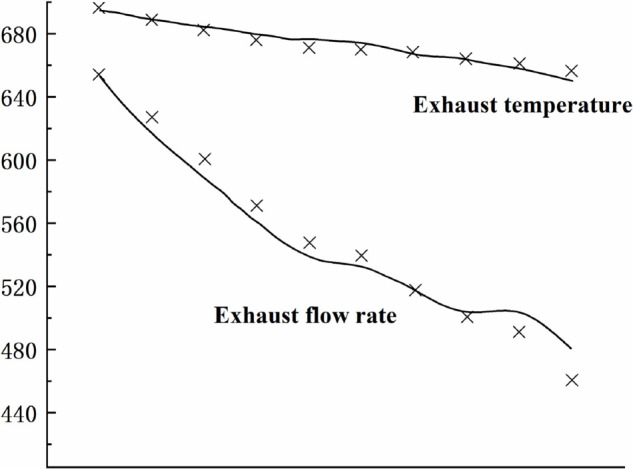
Estimated results when CS=1.062.

From the graph, it can be observed that the modeling method matches the compressor’s experimental data well. when the corrected speed (CS) is in the range of 0.946 to 1.062. At CS = 1.00, the model’s prediction error is the smallest, with the maximum relative error of all predicted values not exceeding 0.2%. The prediction error increases as the speed deviates further from the design speed, with the maximum relative error around 4%. However, for most experimental points, the error is less than 2%, indicating a high prediction accuracy. Therefore, the parameter estimation method is effective, and the characteristic model can accurately represent the compressor’s characteristic information.

## 5 Gas turbine system inverse problem solution

In this section, the study utilizes the compressor model, combustion chamber model and cooling turbine model to establish a comprehensive model of the 9F-class gas turbine as assumed in practical operation. The characteristic curves obtained from modeling are used as the real characteristic curves of this heavy-duty turbine for inverse problem solution, estimating key parameters of each component model. These parameters are then incorporated into the gas turbine model for direct problem solution, and compared with actual operational data of the turbine to validate the accuracy of this approach.

### 5.1 Assumed operational data generation

Parameters of each component and inlet parameters of the turbine are provided in Table 3. Under these settings, performance parameters at design conditions of the turbine are also detailed in the table.

**Table 3:** Assumed operational settings and performance parameters of the actual gas turbine.

Project	Unit	Given Value	GE Company Data
Compressor inlet air mass flow	kg/s	630	623.7
Pressure ratio		15.4	15.4
Compressor isentropic efficiency	%	88	
Combustion efficiency	%	99.5	
Combustor pressure loss	%	3.5	3.5
Combustion temperature	^°^C	1400	
Cooling air parameters	%	18	
Turbine isentropic efficiency	%	87	
Gas turbine power	MW	256	255.6
Gas turbine efficiency	%	35.9	36.9
Turbine exhaust temperature	^°^C	601	
Turbine exhaust flow	kg/s	644	

Combining the measurable parameters of the actual gas turbine, the assumed measurable variables of the actual gas turbine include the following:air flow rate,fuel flow rate,compressor outlet pressure and temperature combustor outlet pressure, combustor outlet temperature, turbine outlet temperature, turbine cooling air inlet temperature, and turbine outlet flow rate. Following the actual experimental process, the experiments of the assumed operational gas turbine are conducted under different environmental temperatures, pressures, and fuel inlet flow rates. Corresponding measurements of various variables are recorded during these experiments.

Inverse problem solving involves the use of maximum likelihood estimation method, combined with assumed experimental data, to infer relevant correction factors in the compressor characteristic equation model and the rated efficiencies of each component (as shown in Table 4).

**Table 4:** List of model parameters to be estimated.

Parameters	symbol
Temperature Flow Correction Factor	*a*_*TFC*_
Pressure Flow Correction Factor	*b*_*PFC*_
Normalized Efficiency Correction Factor	*c*_*NEC*_
Pressure Efficiency Correction Factor	*d*_*PEC*_
Cooling Air Ratio	*F*_*cool*_
Parameters	symbol
Compressor Isentropic Efficiency	$\eta_{C,d}$
Combustion Efficiency	$\eta_{CC}$
Combustion Pressure Loss Coefficient	$\eta_{C,P}$
Turbine Efficiency Correction Factor	$\eta_{T,d}$
Turbine k	*K*

### 5.2 Results of inverse problem solution based on assumed operational data

In this section, using the assumed operational data obtained, the measurement data from the characteristic curves are derived based on the maximum likelihood parameter estimation method, which are used to validate the inverse problem solution method. The estimated values of the relevant parameters based on the simulated data are shown in Table 5.

**Table 5:** Speculative results based on simulated data.

Parameter	Unit	Given Value	Estimated Value	t-value	Relative Errorδ
$\eta_{C,d}$	%	88	87.7	407.1	0.0034
$\eta_{CC}$	%	99.5	99.6	977.8	0.0010
$\eta_{c,p}$		0.035	0.034	44.18	0.0286
*K*		0.237	0.252	17.04	0.0633
*F*_*cool*_	kg/s	113.4	113.93	172.7	0.0047
*a*_*TFC*_			0.2173	6.865	
*b*_*PFC*_			–0.0023	1.15	
*c*_*NEC*_			0.9206	18.83	
*d*_*PEC*_			0.1828	4.18	

As shown in Table 5, the relative errors of the predicted compressor isentropic efficiency, combustor efficiency, and combustor pressure loss coefficient are 0.34%, 0.1%, and 2.86%, respectively. All key parameter errors remained below 3%, and t-test results (α=0.05) confirmed no statistically significant differences between estimated and reference values.

### 5.3 Solving the inverse problem using simulated data with random errors

To test the effectiveness of the maximum likelihood estimation method in estimating the relative standard deviation of measurement errors, random errors following a normal distribution with a constant relative standard deviation were added to the simulated data obtained from modeling (as shown in Table 6). These modified data were used as the simulated experimental data for solving the inverse problem.

**Table 6:** Table of measurement equipment errors.

Measurement Device	Symbol	Relative Standard	Higher Relative Standard Deviation
Air Flow Meter	$\omega_{q_{m,a}}$	0.01	0.02
Flue Gas Flow Meter	$\omega_{q_{m,f}}$	0.01	0.02
High Temperature RTD	$\omega_{T_{h}}$	0.005	0.01
Low Temperature RTD	$\omega_{T_{L}}$	0.003	0.006
Pressure Sensor	$\omega_{p}$	0.005	0.01

To avoid the influence of initial values on the results, the inverse problem was solved by varying the number of experimental data sets and the relative standard deviation used in processing the simulated data, all starting from the same initial values. The results are shown in Table 7.

**Table 7:** List of parameter estimation results corresponding to different simulation experimental data.

Parameter	Initial Value	Relative Std			Large relative Std		
		estimated value	t-value	*δ*	estimated value	t-value	*δ*
$\eta_{C,d}$	88	87.64	250.10	0.0041	87.02	125.20	0.0111
$\eta_{CC}$	95	99.75	347.40	0.0500	99.36	187.80	0.0014
$\eta_{c,p}$	0.02	0.03	18.11	0.6624	0.04	10.73	0.0054
*K*	0.237	0.23	6.60	0.0089	0.24	3.65	0.0242
*F*_*cool*_	100	113.82	51.20	0.1382	115.05	27.05	0.0145
*a*_*TFC*_	1 0.20	2.69		0.15	1.08*		
*b*_*PFC*_	0.9 –0.01	0.26*		-0.05	0.54*		
*c*_*NEC*_	1 0.90	10.12		0.78	4.33		
*d*_*PEC*_	0.5 0.18	2.61		0.07	0.49*		

To avoid the influence of the amount of data, the inverse problem was solved by varying the quantity of experimental data while keeping the initial values and the relative standard deviation constant. The experimental results obtained are shown in Table 8.

**Table 8:** List of parameter estimation results corresponding to different simulation experimental data.

The amount of data	Parameter	Final value	t-value	Reference-t-value	The amount of data	Parameter	Final value	t-value	Reference-t-value
1	$\eta_{C,d}$	88.856	746	1.66846	2	$\eta_{C,d}$	87.88	271.6	1.65576
	$\eta_{CC}$	99.343	1021			$\eta_{CC}$	99.581	841.6	
	$\eta_{c,p}$	0.0507	46.3			$\eta_{c,p}$	0.0343	30.79	
	*K*	0.2530	25.03			*K*	0.2439	13.09	
	*a*_*TFC*_	1.9608	8.615			*a*_*TFC*_	1.8507	3.969	
	*b*_*PFC*_	1.3913	7.436			*b*_*PFC*_	0.03	0.9036*	
	*c*_*NEC*_	0.8476	32.21			*c*_*NEC*_	1.0148	13.61	
	*d*_*PEC*_	0.2408	8.634			*d*_*PEC*_	0.2546	3.364	
3	$\eta_{C,d}$	88.885	953	1.65186	4	$\eta_{C,d}$	89.197	362	1.6499
	$\eta_{CC}$	99.186	836.3			$\eta_{CC}$	99.675	1160	
	$\eta_{c,p}$	0.0615	55.98			$\eta_{c,p}$	0.0541	12.5	
	*K*	0.2670	22.54			*K*	0.2469	21.97	
	*a*_*TFC*_	1.7542	9.531			*a*_*TFC*_	111.159	202.5	
	*b*_*PFC*_	1.0490	8.292			*b*_*PFC*_	0.1962	4.586	
	*c*_*NEC*_	0.8254	39.45			*c*_*NEC*_	0.8473	13.95	
	*d*_*PEC*_	0.2127	11.31			*d*_*PEC*_	0.3588	12.65	

From the Tables 7 and 8, the following observations can be made:

1. Consistency Across Different Simulated Data: The results corresponding to different sets of simulated data show minimal variation. This indicates that the influence of sample randomness is minor, provided that the t-test criteria are satisfied.
2. Accuracy of Estimated Efficiency: The relative error between the estimated values and the originally given values for the rated efficiency of individual components is well below 1%, demonstrating the accuracy of the parameter estimation.
3. Variance Model Estimation: Due to the differences in simulated data derived from the compressor characteristic model and the characteristic curve model, the relative error between the estimated and given values of the variance model is relatively large. The t-value for these estimates is significantly lower than that for other parameters, yet the estimated values generally reflect the relative magnitude of the original given relative standard deviation.
4. Parameter Estimation Accuracy: In Table 8, t-values greater than the reference t-values indicate parameter estimation is accurate. When running two sets of data, the value does not meet the evaluation criteria, suggesting that at Least three sets of data should be provided in practice to ensure the accuracy of experimental results.

Impact of Measurement Error: As the relative standard deviation of measurement errors increases, the t-values of the estimated values decrease, leading to reduced confidence. Therefore, improving measurement accuracy and avoiding large errors are beneficial for enhancing the accuracy of the inverse problem solution.

### 5.4 Comparison with actual operational data

**Fig 9:**
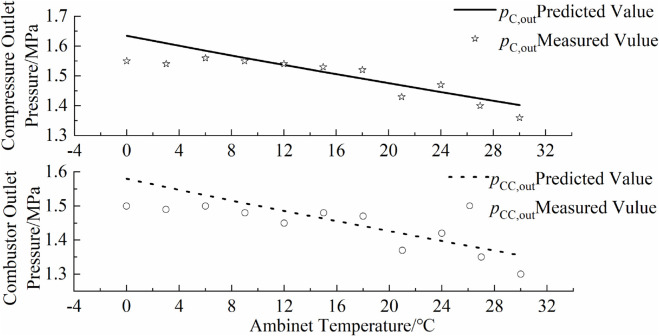
Overlay of predicted and measured pressure.

**Fig 10:**
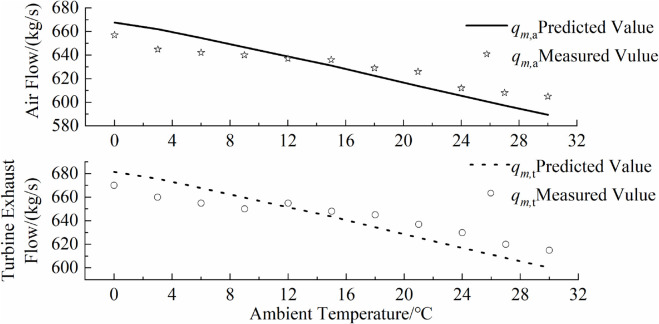
Overlay of predicted and measured flow rates.

**Fig 11:**
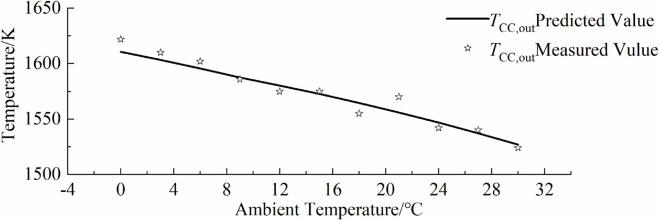
Overlay of predicted and measured low-temperature points.

To verify the accuracy of the inverse problem-solving parameter model, this paper applies the relevant parameters derived from data with larger relative standard deviations to the gas turbine performance prediction model, and compares them with some actual operational measurements. The comparison between the model predictions and actual operational data is shown in Fig 9,10,11,12. As can be seen from the figure, the simulated predictions obtained using the gas turbine characteristic information from the inverse problem-solving method are in good agreement with the actual operational data. It should be noted that although the data used to solve for the gas turbine characteristic parameters in this paper are derived from a hypothetical actual gas turbine, the characteristic curve information reflecting compressor performance is inferred from a certain amount of actual operational data. Additionally, when generating hypothetical actual gas turbine operational data, the influence of sensor measurement errors was also considered, which improves the accuracy of model predictions and verifies the effectiveness of the gas turbine inverse problem-solving process and methods proposed in this paper.

**Fig 12:**
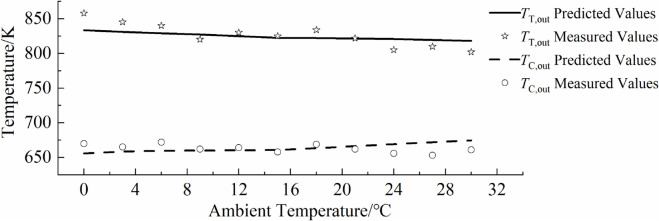
Overlay of predicted and measured high-temperature points.

## 6 Conclusion

This study developed and validated an inverse problem-solving framework that integrates maximum likelihood parameter estimation with a modular modeling methodology, enabling the construction of a mathematical model of a 9FA heavy-duty gas turbine across the full operating regime. Although calibrated and validated using data from a limited number of operating points, the model demonstrated excellent predictive accuracy within the validated range.The proposed method, combining Maximum Likelihood Estimation (MLE) with modular modeling, predicts compressor characteristic curves under steady-state conditions with a maximum relative error of 0.2% for both exhaust flow rate and exhaust temperature. Simulation results show that the relative errors for compressor isentropic efficiency and combustion efficiency, and are 0.34% and 0.1%, with parameter prediction errors below 0.5%. The relative errors for combustion pressure loss coefficient is 2.86% respectively. The t-test results (*α*=0.05) confirmed no statistically significant differences between estimated and reference values. These results indicate that the proposed framework provides a reliable basis for full-condition modeling within the tested operational domain.

The main contribution of this work lies in introducing a new paradigm for constructing full-condition gas turbine models. By combining parameter estimation with modular methodology, the approach substantially reduces the dependence on extensive experimental data, while enabling accurate performance prediction using limited operating data. This methodological advance provides a practical alternative to traditional full-condition modeling strategies.

Future research may extend the model in two directions. First, extrapolation to extreme operating conditions(such as near surge margins or under high back pressure) will be pursued to improve predictive reliability and support early warning for safe operation. Second, efforts will be made to enhance robustness under realistic measurement noise through advanced filtering and uncertainty quantification methods, thereby strengthening the model’s suitability as a core algorithmic component of digital twin systems. Progress in these directions could ultimately enable the framework to evolve into a practical tool for intelligent gas turbine operation and maintenance, supporting applications from real-time condition monitoring to predictive maintenance.

In summary, this research establishes both a theoretical foundation and a practical pathway for developing high-fidelity, data-efficient, full-condition models of heavy-duty gas turbines.

## Supporting information

S1 File Validation by comparison of predicted and measured data. (PDF)
