# Supplementary material for: Solving characteristic parameters of heavy-duty gas turbines using parameter estimation method
Source: PLoS One. 2025 Oct 8;20(10):e0333661. doi: 10.1371/journal.pone.0333661 (PMC12507254; doi:10.1371/journal.pone.0333661)
Supplement: S1 File — (PDF) [file pone.0333661.s001.pdf]

Validation by comparison of predicted and measured data

Validation by comparison of Predicted and Measured Flow Rates

Ambinet temperature/℃	Air flowing (kg/s)		Turbine exhaust flowing (kg/s)	
	$q_{m,a}$ predicted value	$q_{m,a}$ measured vulue	$q_{m,t}$ predicted value	$q_{m,t}$ measured vulue
0	657	667.80	670	681.63
3	645	662.12	660	675.66
6	642	654.67	655	667.90
9	640	646.67	650	659.60
12	637	638.85	655	651.49
15	636	631.22	648	643.558
18	629	622.49	645	634.53
21	626	613.95	637	625.69
24	612	605.60	630	617.03
27	608	597.43	620	608.56
30	605	589.43	615	600.27

This supporting information consists of measured data from the GE 9351FA gas turbine.

Validation by comparison of Predicted and Measured Pressure

Ambinet temperature/ ℃	Compressor outlet pressure/MPa		Combustor outlet pressure/MPa	
	$P_{c,out}$ predicted value	$P_{c,out}$ measured vulue	$P_{cc,out}$ predicted value	$P_{cc,out}$ measured vulue
0	1.63	1.55	1.58	1.5
3	1.61	1.54	1.56	1.49
6	1.58	1.56	1.53	1.5
9	1.56	1.55	1.51	1.48
12	1.54	1.54	1.49	1.45
15	1.51	1.53	1.46	1.48
18	1.49	1.52	1.44	1.47
21	1.47	1.43	1.42	1.37
24	1.45	1.47	1.40	1.42
27	1.42	1.4	1.38	1.35
30	1.40	1.36	1.36	1.3

Validation by comparison of Predicted and Measured Low-Temperature Points

Ambinet temperature/℃	Temperature /K	
	$T_{cc,out}$ predicted value	$T_{cc,out}$ measured vulue
0	1622	1610.71
3	1610	1603.39
6	1602	1595.62
9	1586	1587.40
12	1575	1580.24
15	1575	1572.60
18	1555	1564.47
21	1570	1555.85
24	1542	1546.73
27	1540	1537.10
30	1524	1526.96

Validation by comparison of Predicted and Measured High-Temperature Points

Ambinet temperature/℃	Turbine outlet temperature/K		Compressor outlet temperature/K	
	$P_{c,out}$ predicted value	$P_{c,out}$ measured vulue	$P_{cc,out}$ predicted value	$P_{cc,out}$ measured vulue
0	858	833.67	670	655.94
3	845	831.06	665	658.77
6	840	829.07	672	659.72
9	820	827.17	662	659.98
12	830	825.00	664	660.28
15	825	822.58	658	660.60
18	834	822.25	669	663.50
21	822	821.63	662	666.35
24	805	820.70	656	669.15
27	810	819.46	653	671.89
30	802	817.90	661	674.57
